# Effect on Response Rates of Adding a QR Code to Patient Consent Forms for Qualitative Research in Patients With Cancer: Pilot Randomized Controlled Trial

**DOI:** 10.2196/66681

**Published:** 2025-02-21

**Authors:** Rebecca Wyse, Erin Forbes, Grace Norton, Priscilla Viana Da Silva, Kristy Fakes, Sally Ann Johnston, Stephen R Smith, Alison Zucca

**Affiliations:** 1 School of Medicine and Public Health The University of Newcastle Callaghan, NSW Australia; 2 Hunter Medical Research Institute New Lambton Heights, NSW Australia; 3 Hunter New England Local Health District John Hunter Hospital New Lambton Heights, NSW Australia

**Keywords:** QR code, qualitative research, cancer, randomized controlled trial, RCT, patient recruitment, consent forms, response rates

## Abstract

**Background:**

The successful conduct of health and medical research is largely dependent on participant recruitment. Effective, yet inexpensive methods of increasing response rates for all types of research are required. QR codes are now commonplace, and despite having been extensively used to recruit study participants, a search of the literature failed to reveal any randomized trial investigating the effect of adding a QR code on qualitative research response rates.

**Objective:**

This study aimed to collect data on rates of response, consent, and decline among patients with cancer, and the average time taken to respond following randomization to receive either a QR code or no QR code on the patient consent form for a qualitative research study.

**Methods:**

This was a pilot randomized controlled trial (RCT) embedded within a qualitative research study. In total, 40 eligible patients received a recruitment pack for the qualitative study, which included an information statement, a consent form, and an addressed, stamped envelope to return their consent form. Patients were randomized 1:1 to the control (standard recruitment pack only) or intervention group (standard recruitment pack including modified consent form with a QR code).

**Results:**

In total, 27 out of 40 patients (age: mean 63.0, SD 14.8 years; 45% female) responded to the consent form. A lower proportion of the QR code group (60%) responded (odd ratio [OR] 0.57, 95% CI 0.14-2.37; *P*=.44), compared to 75% of the standard recruitment group. However, a higher proportion of the QR group (35%) consented (OR 1.84, 95% CI 0.41-8.29; *P*=.43), compared to the standard recruitment group (20%). A lower proportion of the QR group (25%) declined (OR 0.34, 95% CI 0.09-1.38; *P*=.13) relative to the standard recruitment group (55%). The mean response time of the QR code group was 16 days (rate ratio [RR] 0.79, 95% CI 0.47-1.35; *P*=.39) compared to 19 days for the standard recruitment group. None of the age-adjusted analyses were statistically significant.

**Conclusions:**

This underpowered pilot study did not find any evidence that offering an option to respond through a QR code on a patient consent form for a qualitative study increased the overall patient response rate (combined rate of consent and decline). However, there was a nonsignificant trend, indicating that more patients who received the QR code consented compared to those who did not receive the QR code. This study provides useful preliminary data on the potential impact of QR codes on patient response rates to invitations to participate in qualitative research and can be used to inform fully powered RCTs.

**Trial Registration:**

OSF Registries 10.17605/OSF.IO/PJ25X; https://doi.org/10.17605/OSF.IO/PJ25X

## Introduction

The successful conduct of health and medical research depends on effective recruitment strategies. Low recruitment rates affect both quantitative and qualitative research designs [1-3], and there is a need to identify effective and affordable methods that are minimally burdensome. For example, a 2019 study reported the costs of 14 recruitment strategies among patients with chronic diseases and reported that 20% of the study budget was spent on recruitment [4]. Given the recent widespread adoption of QR codes [5-8], they offer potential as a valuable and cost-effective recruitment tool. A number of randomized controlled trials (RCTs) have investigated the use of QR codes to recruit participants for quantitative surveys [9]. However, the evidence of the effectiveness of QR codes in increasing survey recruitment is equivocal, with earlier studies [10,11], tending to find no impact of QR codes on response rates, and later studies tending to find a small but significant increases [9,12]. Authors of these studies note that earlier research should be interpreted with caution, given the widespread use and familiarity with QR codes, which is relatively recent [9], and has increased rapidly following the COVID-19 epidemic [13]. A 2024 large RCT (N=5550) that evaluated the effect of including a QR code in a printed and mailed recruitment to a web-based survey found a small but significant increase in survey participation (+1.31%) [12]. It also found that including QR codes resulted in higher participation rates from some harder-to-reach groups, including younger people and single people [12].

However, there is little research about the effect of QR codes on consent rates to other research forms, such as recruitment to research trials or qualitative research, which often require ongoing or more maintained or intense commitment than a one-off survey. A 2024 study randomized recruitment methods ([Twitter rebrand as] X, Facebook [Meta] or QR code displayed on a poster) to a clinical trial and found little to no engagement with the QR code relative to the other recruitment strategies; however, this may have been due to the format (ie, poster) rather than the technology [8].

A literature search failed to identify any study that empirically tests the use of QR codes among people invited to participate in qualitative health research. Given the dearth of studies of this nature, the primary objective of this pilot RCT was to determine whether adding a QR code to a participant consent form for a qualitative research study increased the participant consent rate (ie, the proportion of patients who responded and consented to participate). Secondary objectives were to collect data regarding differences in (1) the proportion of patients who responded (either to consent or decline; this outcome was registered in the Open Science Framework as the proportion of patients who did not respond, but for ease of reporting and understanding, the inverse is reported instead [ie, the proportion who did respond])), (2) the proportion of patients who responded and declined to participate, and (3) the average time (in days) taken to respond measured from the date the recruitment packs were sent.

## Methods

### Study Design

This was a pilot RCT embedded within a qualitative study with patients with colorectal cancer [14]. The purpose of the qualitative study was to determine the views of patients with colorectal cancer about a Digital Health Intervention (“RecoverEsupport”) [15] to enhance their recovery from surgery. The qualitative study required participants to review a newly developed web-based support program and to provide feedback in a recorded telephone-based interview (approximately 20 minutes) that would be scheduled at a time convenient to them. The interview asked them to provide feedback about the relevance and ease of understanding of the program, as well as the information they could recall and the likely impact of the program.

### Participants

Participants were recruited from a Surgical Department at a public hospital in a noncapital metropolitan region of Australia. The Colorectal Cancer Liaison Nurse identified eligible patients from medical records. The eligibility criteria for this pilot RCT were consistent with the eligibility criteria for the qualitative study. Patients were invited to participate in the qualitative study through a recruitment pack containing a printed consent form and a prepaid return envelope (mailed on 1 November 2021). Reminder packs were sent to all nonresponders 3 weeks later (22 November 2021).

For the qualitative study in which the QR code study was embedded, the patient eligibility criteria were as follows: (1) a diagnosis of colorectal cancer, (2) being 18-90 years old, and (3) having undergone a bowel resection for colorectal cancer within the last 6 months (excluding stoma reversals and secondary bowel surgeries).

### Allocation

**Figure 1:**
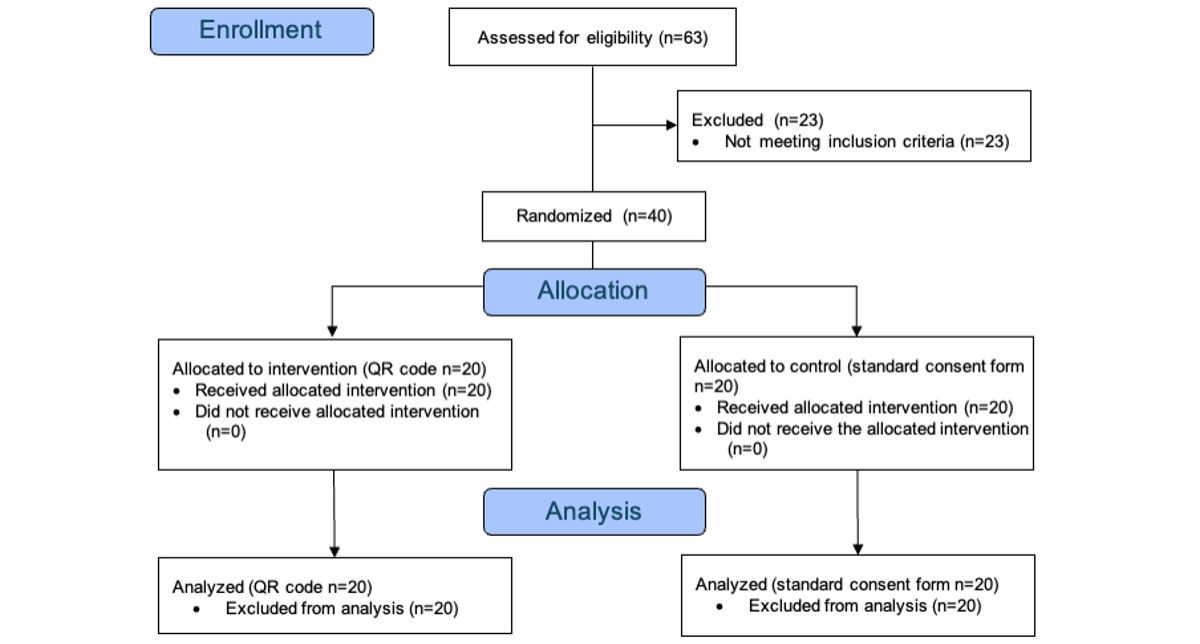
CONSORT Flowchart.

An independent statistician randomly allocated the patients in a 1:1 ratio using a random number function in Microsoft Excel to the intervention or control group (Figure 1).

#### Standard Recruitment (Control)

These patients received the standard consent form (without the QR code). The form included 2 tick boxes for patients to indicate their decision to either consent or decline to participate in the qualitative study.

#### QR Code Group (Intervention)

These patients received an identical consent form to the control group, except this version included a QR code and instructions for accessing the web-based consent form to provide their consent. Scanning the QR code directed the participant to a web-consent-form, identical to the standard consent form, hosted within a REDCap (Research Electronic Data Capture; Vanderbilt University) database. REDCap automatically recorded the date of completion once the patient responded the web-based consent form.

### Outcomes

The primary outcome was the proportion of patients who consented to participate in the qualitative study (through any channel, QR code, or mail). Secondary outcomes included (1) the proportion of patients who responded (either to consent or decline), (2) the proportion of patients who responded and declined to participate in the qualitative study, and (3) the average time in days taken to respond (measured from 1 November 2021 until either the postmark date on the return envelope or the date the web-based consent form was completed).

### Statistical Analysis

Descriptive statistics are presented as counts (%) for categorical variables or mean (SD) for continuous variables. An intention-to-treat approach was used. Unadjusted and age-adjusted logistic regression models were fitted to estimate the odds ratio (OR) and 95% CIs. The between-group difference for the time taken to respond was analyzed using negative binomial instead of Poisson regression modeling (due to overdispersion) and unadjusted and age-adjusted rate ratios (RRs) are presented. Assumptions for the logistic regression model and negative binomial regression model were checked and found to be reasonable.

### Ethical Considerations

The pilot was approved by the Ethics Committees of the Hunter New England Local Health District (2019/ETH00869) and the University of Newcastle (H-2015-0364) and was prospectively registered [16] and is reported in accordance with CONSORT (Consolidated Standards of Reporting Trials) guidelines [17]. Each invited patient received a patient information sheet inviting them to participate in the qualitative research, assuring them that their participation was entirely voluntary and that any information they provided would remain confidential. No incentive was offered for patients to participate. All project staff members were bound by confidentiality agreements. Collected data are stored in a deidentified format.

## Results

### Overview

Of the 63 patients assessed for eligibility, 23 were ineligible, most commonly because they did not have internet access (7/23 patients). The mean age was 58.60 (SD 17.38) years for the QR code, 67.40 (SD 10.35) years for the standard recruitment, totaling 63 (SD 14.8) years for both groups. In total, 45% (n=18) of the sample was female; 40% (n=8) for the QR code and 50% (n=10) for the standard recruitment groups were female. The outcomes are presented below and in Table 1 in sequential order (ie, overall response rate, consent rate, and decline rate).

**Table 1:** Pilot outcomes response rates and time to respond to the invitation to participate in a qualitative research study for participants randomized to receive a quick response code (intervention) or no quick response code (standard recruitment control).

			QR^a^ code (Intervention) (n=20), (%)		Standard recruitment (Control) (n=20) (%)		Unadjusted effect estimate (95% CI)		*P* value		Adjusted for age effect estimate (95% CI)		*P* value
**Responded (overall response rate)^a^**			12 (60)		15 (75)		0.50 (0.13-1.93)		0.3145		0.57 (0.14-2.37)		.44
	Responded and consented (primary outcome)^a^	7 (35)		4 (20)		2.15 (0.52-9.00)		0.2930		1.84 (0.41-8.29)		.43	
	Responded and declined (secondary outcome)^a^	5 (25)		11 (55)		0.27 (0.07-1.04)		0.0577		0.34 (0.09-1.38)		.13	
Response time in days (Secondary outcome)^b^			16.33 (11.12)		19.27 (12.40)		0.85 (0.50-1.43)		0.5361		0.79 (0.47-1.35)		.39

^b^Odds ratio.

^c^Rate ratio.

### Overall Response Rate

A total of 27 patients responded to the consent form, either to consent or decline; 12/20 (60%) from the QR code group and 15/20 (75%) from the standard recruitment group. The unadjusted effect estimate was 0.50 (95% CI 0.13 to 1.93, *P*=.31), indicating that the odds of the intervention group responding were 50% lower compared to the control group. After adjusting for age, the OR was 0.57 (95% CI 0.14 to 2.37, *P*=.44). Although the results suggest a lower overall response rate in the QR code group relative to the standard recruitment group, neither analysis was significant.

### Proportion of Patients Who Responded and Consented (Primary Outcome)

Overall, 11/40 (27.5%) patients consented to participate in the qualitative study. Seven out of 40 consenting patients were from the QR code group (representing 35% of this group), and 4 were from the standard recruitment group (representing 20% of this group). The unadjusted OR was 2.15 (95% CI 0.52-9.00, *P*=.29), indicating that the odds of patients in the intervention group consenting was 2.15 times the odds among control patients, although this was not statistically significant. After adjusting for age, the OR was 1.84 (95% CI 0.41-8.29). However, neither was significant.

### Proportion of Patients Who Responded and Declined to Participate

A total of 16 out of the 40 patients declined to participate in the qualitative study; 5 declining patients were in the QR code group (representing 25% of this group), and 11 were in the standard recruitment group (representing 55% of this group). The unadjusted OR of the QR code group declining to participate was 0.27 (95% CI 0.07-1.04), which was borderline significant (*P*=.06). The age-adjusted OR was 0.34 (95% CI 0.06-1.87, *P*=.21) and was not statistically significant.

### Average Time (in Days) Taken to Respond

The average response time was 16 days (SD 11) days for the QR code group and 19 (SD 12) days for the standard recruitment group. The unadjusted incident RR for the QR code group compared to the standard recruitment group was 0.85 (95% CI 0.5-1.43, *P*=.54), indicating that the response time of the QR code group was 15% lower than the response time for the standard recruitment group. After adjusting for age, the RR was 0.79 (95% CI 0.47-1.35), indicating the QR code group response time was 21% lower than the control group. However, neither was statistically significant.

## Discussion

### Principal Findings

Given the dearth of studies of this nature, the aim of this pilot RCT was to determine the effect on response rates when a QR code was added to a participant consent form for a qualitative research study. Specifically, this pilot investigated the participant response rate, consent rate (primary outcome), rate of decline, as well as the average time taken to respond. Out of 40 patients invited to participate in a qualitative research study were randomized to receive either a standard consent form (control) or a consent form that included a QR code (intervention). The overall response rate (ie, either to consent or decline) was lower in the intervention group relative to the control group, however, the intervention consent rate was higher, the decline rate was lower, and the average time to respond to the invitation to participate was shorter in the intervention group (16 vs 19 days). None of the adjusted estimates of between-group differences were statistically significant. However, our results suggest a trend of patients in the QR code group having higher odds of consenting and lower odds of declining (relative to the control group), but lower odds of responding at all (ie, either to consent or decline). These results should be investigated further in a fully powered RCT.

The overall pattern of results is interesting, despite being nonsignificant. It suggests that the addition of a QR code to a consent form for qualitative research resulted in higher consent rates and lower rates of decline. Both outcomes are desirable for researchers who often spend a large portion of their research budget on recruitment. The shorter response time in the intervention group also suggests that the use of a QR code in recruitment materials could confer benefits to researchers in terms of more timely information about likely recruitment outcomes.

This is the first pilot RCT to investigate the effect of adding a QR code to a consent form within the context of recruitment of patients with cancer to a qualitative study. It is important to consider the rapidly changing context in which QR code research has taken place when comparing these results to the existing literature. Over the past 5 years, responses to the COVID-19 pandemic have made QR code levels of awareness, familiarity, and skill in the community is now much higher. The more recent RCTs testing the addition of QR codes to surveys, showed small but significant increases, of approximately 1-2% in consent rates [9,12]. The magnitude of the changes observed in the current pilot are much greater (15% increase in consent rates), despite being nonsignificant due to under-powering. This may be due to the targeted nature of the study (ie, people who had previously had colorectal cancer surgery, compared with a general population statewide public health survey) [12] and due to differences in the specific nature of what they were being asked to do (ie, review an intervention designed to support patients with colorectal cancer going through surgery and provide feedback in a 1:1 interview, vs complete a web-based public health survey).

The randomized design is a strength; however, a few limitations should be considered when interpreting the findings of this term. Despite the randomization, due to the small sample size, the groups were not evenly distributed in age, which was subsequently adjusted for. The low sample size is another limitation, although it is still within the recommended range for pilot studies [18]. Therefore, as a next step, a fully powered RCT is recommended. Furthermore, the study did not collect data about whether the participants actually used the QR to respond (only overall response rates) or the characteristics of those users who responded using this channel. This would be valuable information to collect and analyze in future studies to better understand the impact of QR codes on the diversity of recruited participants. Given the context in which this research occurred, it is unclear the extent to which these findings would generalize to the general population, younger people or both groups. Future RCTs should also allow for subgroup analysis to determine the specific effects of QR codes on recruiting different age groups.

### Conclusion

Despite the nonsignificance of the findings in this underpowered pilot trial, this research question warrants further investigation. Given their ease of use and inexpensive application, using QR codes on consent forms may hold promise for improving recruitment to qualitative research studies. As recruitment often consumes a large proportion of research budgets, even a small percentage of change could have important benefits for researchers and research programs. QR codes may be a low-risk strategy to complement other attempts to maximize research response rates, although a fully powered RCT is required to confirm this. Further research using larger and more diverse samples is warranted. This pilot study provides important preliminary data on which such studies could be based.

## Appendix

Multimedia Appendix 1 CONSORT (Consolidated Standards of Reporting Trials) extension for pilot and feasibility studies checklist.

## Abbreviations

CONSORT: Consolidated Standards of Reporting Trials
OR: odds ratio
RCT: randomized controlled trial
REDCap: Research Electronic Data Capture
RR: rate ratio

## Data Availability

The datasets generated during this study or analysis of the data are available from the corresponding author on reasonable request and pending ethical approval.
